# Amphetamine Abuse Related Acute Myocardial Infarction

**DOI:** 10.1155/2016/7967851

**Published:** 2016-02-21

**Authors:** Archana Sinha, O'Dene Lewis, Rajan Kumar, Sri Lakshmi Hyndavi Yeruva, Bryan H. Curry

**Affiliations:** ^1^Division of Cardiology, Saint Luke's University Health Network, 801 Ostrum Street, Bethlehem, PA 18015, USA; ^2^Division of Pulmonary Medicine, Howard University Hospital, 2041 Georgia Avenue, Washington, DC 20060, USA; ^3^Division of Hematology-Oncology, Howard University Hospital, 2041 Georgia Avenue, Washington, DC 20060, USA; ^4^Division of Cardiology, Howard University Hospital, 2041 Georgia Avenue, Washington, DC 20060, USA

## Abstract

Amphetamine abuse is a global problem. The cardiotoxic manifestations like acute myocardial infarction (AMI), heart failure, or arrhythmia related to misuse of amphetamine and its synthetic derivatives have been documented but are rather rare. Amphetamine-related AMI is even rarer. We report two cases of men who came to emergency department (ED) with chest pain, palpitation, or seizure and were subsequently found to have myocardial infarction associated with the use of amphetamines. It is crucial that, with increase in amphetamine abuse, clinicians are aware of this potentially dire complication. Patients with low to intermediate risk for coronary artery disease with atypical presentation may benefit from obtaining detailed substance abuse history and urine drug screen if deemed necessary.

## 1. Introduction

Amphetamine is a potent central nervous system stimulant and a substance of widespread abuse. United Nations Office on Drugs and Crime ranks amphetamine-type stimulants such as MDMA (popularly known as Ecstasy or Molly) and methamphetamine as the world's second most widely used type of drug after cannabis [1]. According to the figures released by National Survey on Drug Use and Health, 1.4 million or 0.5 percent of individuals aged 12 years or older were nonmedical users of stimulants in 2013 [2]. The use of amphetamine appears to vary with gender and race. It is predominantly seen in unemployed single white men aged 20–35 years [3]. Data suggests that Caucasians use amphetamines more than African Americans [4]. The role of cocaine as a causative agent of AMI is well established [5, 6] but AMI due to amphetamine use or abuse has not been as widely recognized. Westover et al. showed modest, though statistically significant, association between amphetamine abuse and AMI in their population-based study [7]. The most popular amphetamines encountered in abuse include amphetamine, 3,4-methylenedioxymethamphetamine (MDMA), 4-methoxyamphetamine (PMA), 3,4-methylenedioxyamphetamine (MDA), 3,4-methylenedioxyethylamphetamine (MDEA), and 4-methylthioamphetamine (4-MTA) [8]. We report two cases of previously healthy men who presented with atypical chest pain and palpitations to the ED and later were diagnosed with AMI associated with use of amphetamines.

## 2. Case  1

**Figure 1:**
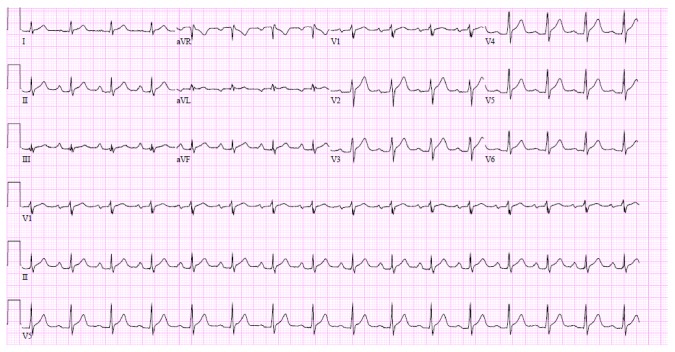
Sinus tachycardia and diffuse ST segment elevation suggestive of early repolarization.

A 41-year-old African American male was brought to our ED with worsening constant, central, pleuritic chest pain of one-day duration that woke him from sleep. He also complained of nausea, tingling, and numbness of both hands. He reported a history of few weeks of intermittent chest pain in the same location, with no aggravating or relieving factors. His past medical history was significant for dyslipidemia. He denied prescription or illicit drug use but gave a remote history of smoking, alcohol, and marijuana abuse. He had no known drug allergy. His father passed away from a myocardial infarction at 66 years of age. On presentation to the ED his blood pressure was 107/60 mm Hg, heart rate was 78 beats per minute, respiratory rate was 16 per minute, and temperature was 97°F with an oxygen saturation of 99% on room air. Cardiovascular examination was unremarkable. His lungs were clear to auscultation and his abdominal examination was within normal limits. Electrocardiogram (EKG) showed sinus tachycardia with diffuse ST segment elevation suggestive of early repolarization (Figure 1). His initial set of cardiac enzymes showed markedly elevated CPK of 2176 U/L, CK-MB of 55.5 ng/mL, troponin I of 2.22 ng/mL, and myoglobin of 467 ng/mL (Table 1). He was admitted to the cardiac unit for hemodynamic monitoring, serial serum cardiac enzymes levels assays, serial EKGs, and assessment of left ventricular function. The rest of his laboratory values are listed in Table 2. The urine drug screen was positive for amphetamines and negative for cocaine, opiates, barbiturates, cannabinoids, benzodiazepines, and phencyclidine, methadone, and propoxyphene. Chest X-ray showed no acute pathology. A transthoracic echocardiogram showed normal left ventricular systolic function without any regional wall motion abnormalities. Cardiac catheterization revealed normal epicardial coronary arteries without any evidence of atherosclerotic disease. Serum troponin levels showed a downward trend on follow-up lab tests. The patient was started on amlodipine 10 mg for possible coronary vasospasm. He was discharged home after two days of hospitalization. The final diagnosis of AMI likely secondary to amphetamine-induced vasospasm was made.

## 3. Case  2

**Figure 2:**
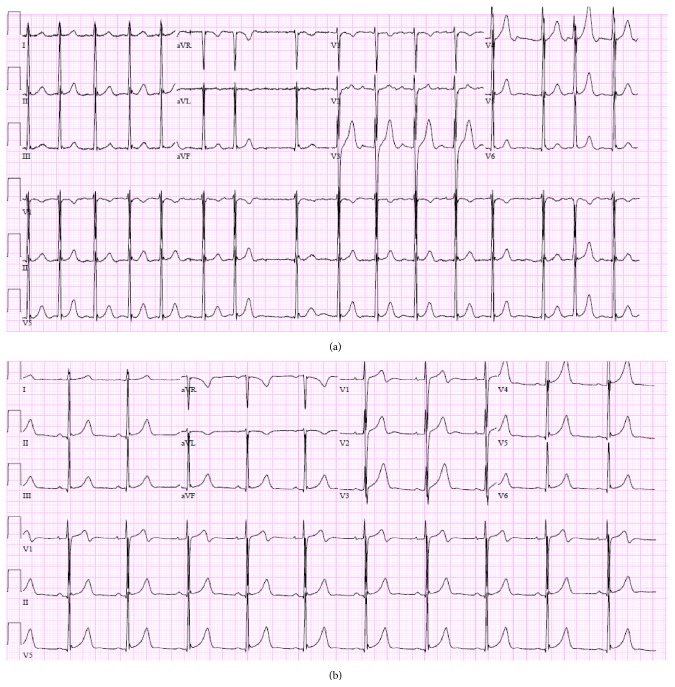
(a) Atrial fibrillation without any significant ST changes. (b) Normal sinus rhythm with early repolarization.

A 23-year-old African American male was brought to our ED with a seizure episode preceded by palpitations and diaphoresis. He was on a rigorous exercise regimen and had started taking an over-the-counter product called Shakeology one week before. At the time of admission, his blood pressure was 101/64 mm Hg, heart rate was 154 beats per minute, respiratory rate was 18 per minute, and temperature was 98°F with an oxygen saturation of 98% on room air. He denied prescription or illicit drug use but on the day of presentation he did drink some alcohol. He admitted to smoking two cigarettes per week and having a couple of beers on the weekend. His past medical history was unremarkable and he had no known drug allergies. There was no significant family history of any cardiac conditions. Cardiovascular examination revealed an irregularly irregular heart beat and the EKG showed atrial fibrillation without any significant ST changes (Figure 2(a)). His lungs were clear to auscultation and abdominal examination was within normal limits. His initial cardiac enzymes were elevated with CPK of 271 U/L, CK-MB of 29.4 ng/mL, troponin I of 1.73 ng/mL, and myoglobin of 79 ng/mL (Table 1). He was admitted to cardiac unit for further monitoring and management. Repeat EKG showed spontaneous conversion to normal sinus rhythm (Figure 2(b)). The rest of his laboratory values were normal (Table 2). Troponin I levels peaked at 9.57 ng/mL. No acute pathology was noted on chest X-ray. Cardiac CT angiogram with calcium scoring was done. It showed calcium score of 0 Agatston units and no evidence of coronary artery disease. The urine drug screen was positive for amphetamines and negative for cocaine, opiates, barbiturates, cannabinoids, benzodiazepines, and phencyclidine, methadone, and propoxyphene. Coronary vasospasm likely secondary to amphetamine was considered possible etiology and he was started on amlodipine 10 mg. He was discharged home on fourth day after resolution of his symptoms. The final diagnosis of atrial fibrillation and non-ST segment elevation myocardial infarction likely secondary to amphetamine-induced vasospasm was made.

## 4. Discussion

Amphetamine is a synthetic derivative of phenethylamine. Amphetamines are central nervous system stimulants that stimulate the release of catecholamines especially norepinephrine and dopamine from presynaptic nerve endings and prevent their reuptake, thus creating a hyperadrenergic state [9]. Its history of abuse dates back to its development more than 100 years ago. The last twenty-five years have seen a rapid surge in illicit use of amphetamine and related compounds in the United States. The routes of administration can be oral, intravenous (IV), and inhalation. With oral use there is approximately 1-hour delay in symptoms, whereas when inhaled or injected effects are seen within few minutes. The peak plasma concentration is achieved in 5 minutes with IV use and in 2 to 3 hours after ingestion. The rate of metabolism is highly variable and up to 30% of the parent compound can be excreted unchanged in the urine. Plasma half-life ranges from 5 to 30 hours depending on urine flow and pH [10].

The most common cardiovascular side effects of amphetamine abuse are hypertension and tachycardia [11]. Other common complaints can be chest pain, palpitations, and shortness of breath. There have been reports of serious cardiovascular complications like cardiomyopathy, cardiac dysrhythmia, myocardial infarction, cor pulmonale, myocarditis, necrotizing vasculitis, coronary rupture, and sudden cardiac arrest [11–14]. The exact pathophysiological mechanism of myocardial infarction following amphetamine use is unclear. Proposed mechanisms include coronary vasospasm, coronary spasm with intracoronary thrombus, prothrombotic activation [15, 16], increased myocardial oxygen demand induced by catecholamines [17], and catecholamine-mediated platelet aggregation with subsequent thrombus formation [18]. The role of amphetamine in inducing vasoconstriction and vasospasm is not completely explained by adrenergic stimulation [19].

Our patients had no therapeutic indication for amphetamine-like substances and denied any recreational drug use. The exact route of intake could not be ascertained. 45% of the adults in a recent single centre study by Alghamdi et al. did not admit to substance abuse. Adults aged <40 years were more likely to admit to substance abuse [20]. Urine drug screens are generally performed using either immunoassays or gas chromatography-mass spectrometry (GC-MS) [21]. One major problem with immunoassays is false-positive results. Therefore, a more specific confirmatory test such as GC-MS is needed to confirm a positive finding with an immunoassay. GC-MS is more accurate than an immunoassay but it is more expensive and time-consuming [22]. Our patients were not on any drugs or dietary supplements that are known to cause false-positive immunoassay [23]. One of our limitations is absence of GC-MS urine drug screen or serum amphetamine levels for the patients.

Patient 2 had history of consumption of a meal replacement shake (Shakeology). Popular dietary supplements are known to cause false-positive urine drug screen for amphetamine [23, 24] and also have been implicated in causing sudden cardiac death and AMI [25, 26]. Shakeology is not mentioned in the list of such dietary supplements released by the concerned agencies [27].

Patient 1 had normal sinus rhythm with early repolarization and patients 2 had atrial fibrillation with nonspecific ST changes. Serial cardiac biomarkers were elevated in both patients. Liebetrau et al. proposed diagnostic approach for AMI in patients with atrial fibrillation and symptoms suggestive of AMI. The specificity-optimized cut-off values higher than the 99th percentile concentration (0.032 ng/mL) and additional use of the 3-hour change in troponin concentration lead to a positive predictive value of more than 95%, facilitating identification of patients with AMI [28]. Patient 2 had atrial fibrillation that lasted for few minutes and resolved spontaneously. The maximal noted heart rate was 154 beats per minute. He did not have any evidence of hypotension. His troponin I on presentation was 1.73 ng/mL and subsequently increased to 9.57 ng/mL four hours later. It does not seem plausible that atrial fibrillation that was transient and without any hemodynamic compromise would lead to greater than fivefold increase in troponin on subsequent testing.

The treatment of AMI attributed to amphetamine abuse is not clearly defined [29, 30]. Calcium channel blockers may play an important role in the treatment of AMI due to amphetamines and as in our cases they may be effective in treatment of suspected coronary vasospasm. Beta-adrenergic receptor blocker administration should be avoided until the pathophysiology of this condition has been clarified as they may exacerbate coronary vasospasm. Thrombolytic therapy or intravenous anticoagulants may be used if coronary thrombus is present [19].

Patient 1 had cardiac catheterization while Patient 2 had CT coronary angiography given his low risk for coronary artery disease. No evidence of coronary artery disease was found in either subject. Both patients had good outcome and were discharged home to follow-up in cardiology clinic.

## 5. Conclusion

Amphetamine and related substances are now among the most commonly abused drugs worldwide. Amphetamine associated AMI may become more common in the acute care setting if the rate of amphetamine abuse continues to increase. Although the exact mechanism by which it does so is still unclear, there seems to be intricate interaction of host and drug factors that is responsible for the variability in clinical presentation and outcome. Clinically a number of areas need to be addressed, the most important being the appropriate therapeutic approach. Physicians working in the emergency departments as well as cardiologists should be sensitive to this complication, as early diagnosis can be the key to successful management of this potentially fatal complication.

## Figures and Tables

**Table 1:** Laboratory data of cardiac enzyme trends.

Cardiac enzymes	Case 1			Case 2		
	1st set	2nd set	3rd set	1st set	2nd set	3rd set
CPK (35–230 IU/L)	2176	2174	4895	271	481	365
CK-MB (0.0–5.0 ng/mL)	55.5	40	9.7	29.4	56.5	23.8
Troponin (0.00–0.03 ng/mL)	2.22	1.3	0.31	1.73	9.57	6.74
Myoglobin (0–450 pg/mL)	467	139	287	79	55	19

CPK: creatine phosphokinase; CK-MB: creatine kinase (CK) MB isoenzyme.

**Table 2:** Laboratory data of the patients at admission in the hospital and during hospitalization.

Laboratory parameter (normal range)	Case 1	Case 2
Urea (7–25 mg/dL)	9	21
Creatinine (0.6–1.2 mg/dL)	0.9	1.5
Sodium (135–148 mEq/L)	137	137
Potassium (3.5–5.3 mEq/L)	4.0	3.5
Calcium (8.5–10.5 mg/dL)	9.1	9.0
Phosphorus (2.5–4.5 mg/dL)	2.9	4.7
Magnesium (1.7–2.5 mg/dL)	2.0	2.4
Prothrombin time (12.5–14.5 seconds)	13.0	14.8
Partial thromboplastin time (24–34 seconds)	31.5	31.6
International normalized ratio (1.12–1.46)	0.99	1.17
White blood cell (3.2–10.6) cells/liter	8	10.5
Hemoglobin (12.1–15.9 g/dL)	14.9	12.6
Mean corpuscular volume (77.8–94.0 fL)	87	91.9
Platelet (177000–406000/*μ*L)	161	227
Erythrocyte sedimentation rate (0–10 mm/h)	20	—
Thyroid stimulation hormone (0.4–40 mU/mL)	—	3.14
Human immune deficiency virus	Nonreactive	Nonreactive
Total cholesterol levels (145–200 mg/dL)	199	150
Serum triglycerides (20–160 mg/dL)	151	102
LDL cholesterol (50–150 mg/dL)	135	99
HDL cholesterol (45–85 mg/dL)	30	46
Fasting blood sugar (60–110 mg/dL)	171	100

LDL: low-density lipoprotein; HDL; high-density lipoprotein.
